# Predicting changes in molluscan spatial distributions in mangrove forests in response to sea level rise

**DOI:** 10.1002/ece3.9033

**Published:** 2022-07-13

**Authors:** Wei Ma, Mao Wang, Haifeng Fu, Chaoyi Tang, Wenqing Wang

**Affiliations:** ^1^ Key Laboratory of the Coastal and Wetland Ecosystems (Xiamen University) Ministry of Education, College of the Environment and Ecology Xiamen University Xiamen China

**Keywords:** dynamic model, mangrove, mollusk, sea level rise, species zonation, surface elevation change

## Abstract

Mollusks are an important component of the mangrove ecosystem, and the vertical distributions of molluscan species in this ecosystem are primarily dictated by tidal inundation. Thus, sea level rise (SLR) may have profound effects on mangrove mollusk communities. Here, we used dynamic empirical models, based on measurements of surface elevation change, sediment accretion, and molluscan zonation patterns, to predict changes in molluscan spatial distributions in response to different sea level rise rates in the mangrove forests of Zhenzhu Bay (Guangxi, China). The change in surface elevation was 4.76–9.61 mm year^−1^ during the study period (2016–2020), and the magnitude of surface‐elevation change decreased exponentially as original surface elevation increased. Based on our model results, we predicted that mangrove mollusks might successfully adapt to a low rate of SLR (2.00–4.57 mm year^−1^) by 2100, with mollusks moving seaward and those in the lower intertidal zones expanding into newly available zones. However, as SLR rate increased (4.57–8.14 mm year^−1^), our models predicted that surface elevations would decrease beginning in the high intertidal zones and gradually spread to the low intertidal zones. Finally, at high rates of SLR (8.14–16.00 mm year^−1^), surface elevations were predicted to decrease across the elevation gradient, with mollusks moving landward and species in higher intertidal zones blocked by landward barriers. Tidal inundation and the consequent increases in interspecific competition and predation pressure were predicted to threaten the survival of many molluscan groups in higher intertidal zones, especially arboreal and infaunal mollusks at the landward edge of the mangroves, resulting in a substantial reduction in the abundance of original species on the landward edge. Thus, future efforts to conserve mangrove floral and faunal diversity should prioritize species restricted to landward mangrove areas and protect potential species habitats.

## Graphical abstract

The current predictable sea‐level rise would not lead to the total loss of mangrove in a subtropical estuarine wetland in southwestern China, but it could alter molluscs zonation along the intertidal profile. Rapid sea‐level rise and the consequent coastal squeeze threatened the survival of many molluscan groups in higher intertidal zones, especially species at the landward edge of the mangroves.

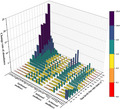

## 1 INTRODUCTION

Mangroves, which are coastal forests that inhabit the intertidal regions of the tropics and subtropics worldwide (Alongi, 2009), provide suitable reproductive and nursery habitats for many benthic fauna (Nagelkerken et al., 2008). In addition to providing shelter, mangrove plants export significant amounts of organic matter, helping to maintain high levels of biodiversity and providing important ecological and economic services (Barbier et al., 2011; Lee et al., 2014). Mangroves are particularly threatened by accelerated sea level rise (SLR) because they are restricted to a relatively narrow elevation zone in the intertidal region (Gilman et al., 2008; Gopal, 2013; Lovelock et al., 2015). Observed and anticipated future rates of SLR are concerning, as SLR may threaten the stability of mangrove habitats and the ecological services they provide (Phan et al., 2014; Woodroffe et al., 2016). It is unclear whether mangroves will adapt to the anticipated impacts of SLR and successfully maintain ecological function (Friess et al., 2020).

The conservation of molluscan diversity is important for the maintenance of ecological functions in mangroves (Lee et al., 2014; Leung, 2015). Mollusks are one of the most abundant and conspicuous macrofauna in mangroves, occupy a wide range of ecological niches (MacNae, 1968; Nagelkerken et al., 2008), and influence mangrove community structure by feeding on the seeds of mangrove plants (Bosire et al., 2008; Fratini et al., 2004; Smith III et al., 1989). Mollusks play an important role in the decomposition and transfer of mangrove organic matter by feeding on sediments and plant matter, and provide a source of food for vertebrate predators, such as birds and fishes (Nagelkerken et al., 2008; Peng et al., 2017). Therefore, mollusks may strongly influence the biogeochemical processes and trophodynamics of mangrove forests (Lee, 2008; Peng et al., 2017).

Species distributions in intertidal habitats are largely controlled by tidal inundation, and the resulting zonation patterns are ubiquitous across both mangrove and mollusk taxa (Crase et al., 2013; Leong et al., 2018; Ma et al., 2020; Watson, 1928). SLR increases the frequency and duration of inundation beyond species‐specific physiological thresholds, thereby altering the distribution of species in the intertidal zone (Ball, 1988; Friess et al., 2012). The effects of SLR on the distribution of mangrove plants have attracted widespread attention (Lovelock et al., 2015; Woodroffe et al., 2016). In addition, several studies have predicted changes in the distributions of mollusks on intertidal mudflats and seagrass beds in response to SLR (Birchenough et al., 2015; Fujii & Raffaelli, 2008; Singer et al., 2017). However, few studies have focused on the response of mangrove mollusks to SLR. Mangrove mollusks can be divided into arboreal, epifaunal, and infaunal species, based on the habitat they occupy (Salmo et al., 2017). Mollusks in each assemblage may respond differently to SLR because coping strategies in response to tidal inundation may be habitat‐dependent (Ma et al., 2020).

Mangroves that are unable to maintain their relative position in the intertidal zone (i.e., via landward migration to higher elevations) due to natural or artificial topographical barriers adapt to SLR primarily by raising the sediment surface itself, which may include the accumulation of organic matter derived from roots and sediment accretion on the soil surface (Cahoon & Hensel, 2006; Kirwan & Murray, 2007; Lovelock et al., 2015; McKee et al., 2007). In particular, sediment accretion plays a crucial role in the Indo‐Pacific due to the high sediment levels in this region (Lovelock et al., 2015). Sediment accretion and surface elevation changes in mangrove forests depend mainly on vegetation type and the duration of inundation (Crase et al., 2013; Krauss et al., 2003; Krauss et al., 2017). Mangrove plants encourage surface level increase by reducing water flow through above‐ground structures such as aerial roots, thus increasing sediment accretion (Horstman et al., 2015; Krauss et al., 2014; Kumara et al., 2010). Mangrove species zonation leads to differences in sediment accretion and rates of surface level change among elevations (Krauss et al., 2003) Accretion rates also vary among elevations due to the associated differences in inundation duration: Accretion rate generally increases nonlinearly with increasing inundation duration (Kirwan et al., 2016; Kolker et al., 2010). Accretion rates should thus be monitored in the context of elevation gradients. However, to date, most monitoring studies of accretion rates have compared accretion rates along horizontal, rather than vertical, gradients (Bomer et al., 2020; Lane et al., 2020).

The main goal of this study was to evaluate the effects of different SLR rates on mangrove mollusk communities. We performed quadrat mollusk sampling along a surface elevation gradient in Zhenzhu Bay (Guangxi, China) to investigate the relationship between molluscan zonation and surface elevation. The rod surface elevation table‐marker horizon methodology (RSET‐MH) (Cahoon et al., 2002) was used to measure surface elevation changes and sediment accretion along the elevation gradient. Dynamic models were introduced to predict surface elevation changes at different vertical positions in response to various rates of SLR, considering the ecogeomorphic feedback between tidal inundation and sediment accretion. Finally, the surface elevation changes predicted by the models were combined with the relationship between molluscan zonation and surface elevation to predict changes in molluscan spatial distributions in response to SLR.

## 2 METHODS

### 2.1 Study site

**FIGURE 1:**
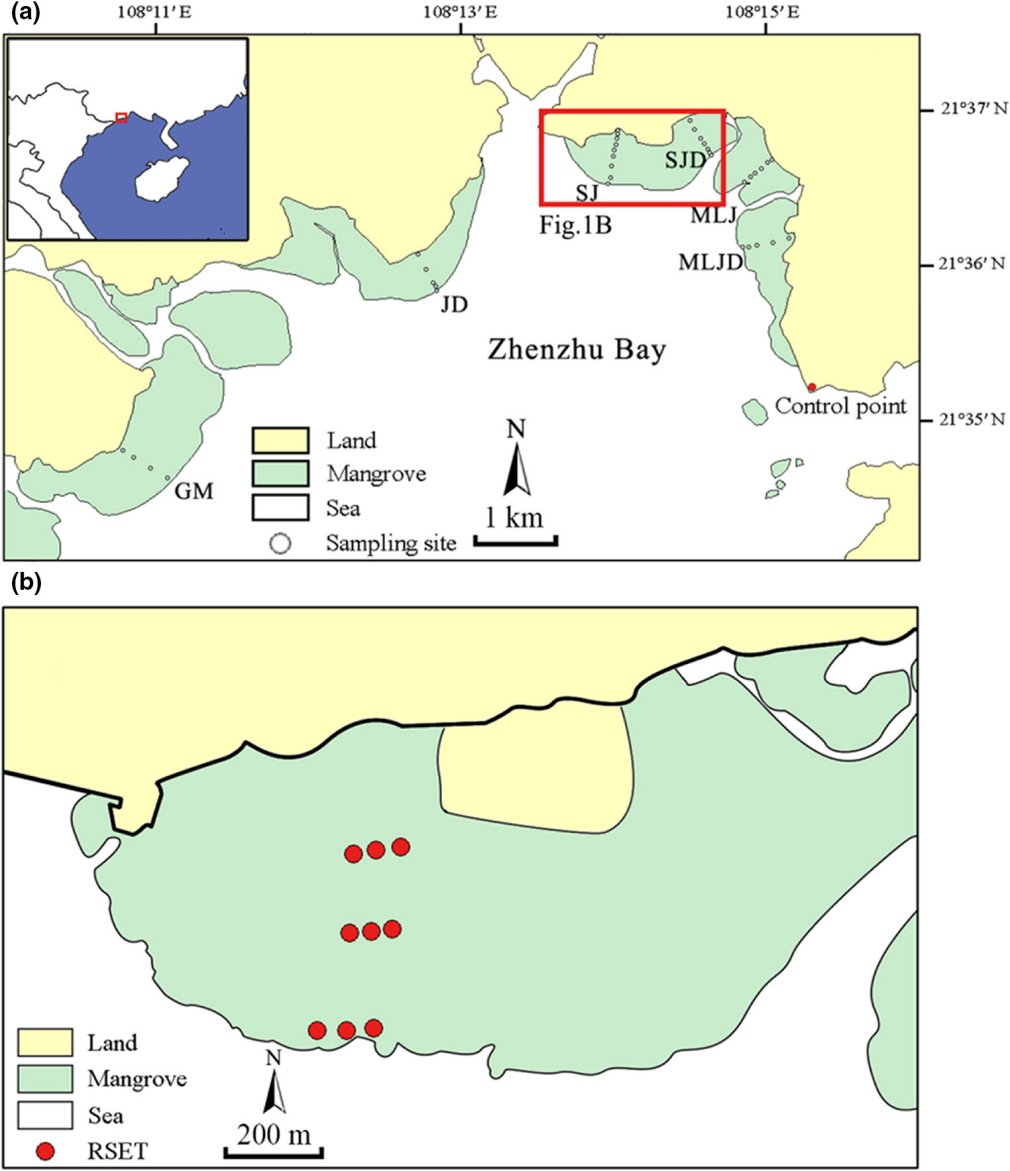
(a) Map showing control point (red dot) and sampling sites (white dots) of the six transects through the mangrove forests of Zhenzhu Bay, Guangxi, China. (b) Enlargement of the red box in panel a, showing rod surface elevation table (RSET) benchmarks (red dots)

This study was carried out at Zhenzhu Bay in Beilun Estuary National Nature Reserve, Guangxi, China, which is the most southwestern national mangrove reserve on the coast of mainland China (Figure 1a). Zhenzhu Bay is a sheltered, funnel‐shaped bay in the South China Sea that is bordered by 17.33 km^2^ of mangrove forests (Wang & Wang, 2007). The bay has a subtropical monsoon climate, with a mean annual temperature of 22.5°C and a mean annual rainfall of 2220 mm. Tides in the bay are diurnal, and the average tidal range is 2.24 m (EBCBS, 1993). The bay is fed by the Jiangping and Huangzhu Rivers, which provide a stable supply of sediments (Li & Zhou, 2017). Mangrove plants in the bay exhibit obvious zonation patterns, with *Aegiceras corniculatum*, *Avicennia marina*, *Kandelia obovata*, and *Bruguiera gymnorhiza* found lowest to highest along the elevation gradient (Ma et al., 2020). There are artificial or natural topographical barriers, such as seawalls, roads, and mountains, at the landward edge of 85% of the mangrove forests in the bay, potentially leading to coastal squeeze (Fan & Li, 1997).

### 2.2 Topographical field survey

Six transects were drawn from the seaward forest edge to the landward forest edge, and elevation was measured in 5–10 m horizontal intervals along each transect using a Global Navigation Satellite System‐Real Time Kinematic GPS unit (G970 GNSS RTK, UniStrong Inc.) with a vertical precision of 15 mm (Figure 1a). Measured elevations were converted to elevations above/below local mean sea level, which was determined based on control points located 3 km from the study area (Figure 1a). To sample along the entire elevation gradient, a total of 36 sampling sites were established at 25 cm intervals between −15 and 150 cm elevation; there was no sampling site at 135 cm as there was a gap in the mangrove forest at this elevation (Table 1).

**TABLE 1:** Distance of each sampling site from the seaward forest edge and its elevation with respect to sea level

Sample site	Distance from the seaward forest edge (m)	Surface elevation (cm)	Sample site	Distance from the seaward forest edge (m)	Surface elevation (cm)
SJ‐1	14.80	−15	MLJD‐3	160.84	60
SJ‐2	47.68	10	MLJD‐4	372.88	60
SJ‐3	151.00	35	MLJD‐5	597.25	35
SJ‐4	405.23	60	MLJ‐1	6.71	−15
SJ‐5	481.18	35	MLJ‐2	25.70	10
SJ‐6	557.18	60	MLJ‐3	129.83	35
SJ‐7	634.54	85	MLJ‐4	215.12	60
SJ‐8	656.09	110	MLJ‐5	357.09	60
SJ‐9	680.03	150	MLJ‐6	386.54	85
SJD‐1	12.42	−15	JD‐1	8.74	−15
SJD‐2	25.34	10	JD‐2	24.60	10
SJD‐3	55.41	35	JD‐3	102.12	35
SJD‐4	144.25	60	JD‐4	305.75	60
SJD‐5	255.40	60	JD‐5	582.87	35
SJD‐6	360.02	35	GM‐1	21.68	35
SJD‐7	473.35	10	GM‐2	209.69	60
MLJD‐1	5.37	10	GM‐3	469.62	85
MLJD‐2	92.61	35	GM‐4	641.94	110

### 2.3 Molluscan sampling

From April 2017 to January 2018, mollusks were sampled quarterly at each of the 36 sampling sites during low tide. To collect mollusks as comprehensively as possible, we sampled the arboreal, epifaunal, and infaunal molluscan communities within the mangrove forest at each site. Arboreal mollusks attached to the mangrove trunks, leaves, and prop roots were collected by hand in three randomly placed quadrats (5 × 5 m; 10 m apart) at each sampling site. To collect epifaunal mollusks, five quadrats (1 × 1 m; 5 m apart) were randomly placed at each sampling site, and all epifaunal mollusks on the sediment surfaces within each quadrat were collected. To collect infaunal mollusks, one quadrat (0.25 × 0.25 m) was randomly placed in each epifaunal quadrat. The sediment in each infaunal quadrat was collected to a depth of 30 cm and sieved through a 1 mm mesh to obtain infaunal mollusks. All specimens were identified to species using Okutani (Okutani, 2000) and Wang (Wang et al., 2016), and then counted and weighted.

### 2.4 Surface elevation change and sediment accretion

#### 2.4.1 Changes in surface elevation

Changes in surface elevation were recorded using nine RSET instruments (Cahoon et al., 2002) at 3–12 month intervals between July 2016 and August 2020, comprising a total of eight sets of measurements; the total period assessed was 49 months. The nine RSET benchmarks were established in July 2015 and distributed among the *A. corniculatum*, *K. obovata*, and *B. gymnorhiza* communities along the SJ transect at depths of 5–6 m (Figure 1b). Each RSET instrument consisted of a deep benchmark and a measuring arm. During RSET installation, a deep benchmark was established at each measurement location by driving stainless steel rods (15 mm in diameter) into the soil profile until refusal and then fixing the rods in place with cement. A machine‐notched pipe was connected to the top of the stainless‐steel rod to hold the measuring arm. When it was time to take a reading, the measuring arm was attached to the benchmark and leveled. Nine fiberglass pins were inserted into the measuring arm and lowered onto the soil surface, and the distance from the arm to the top of each pin was measured. These nine distance measurements were repeated in four directions, yielding 36 total measurements, which were combined to generate an average reading per sampling site.

**FIGURE 2:**
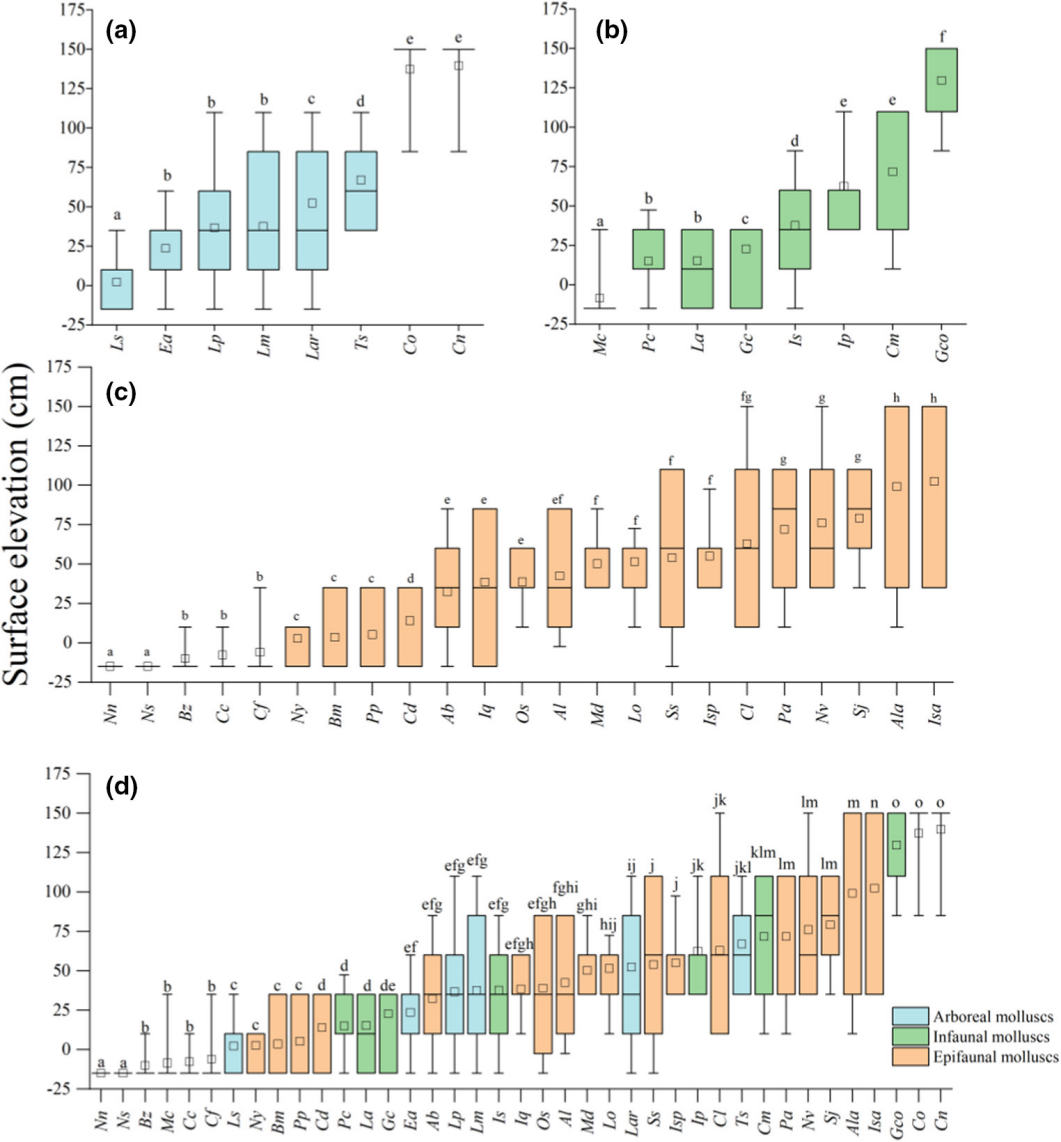
Vertical distribution of (a) arboreal mollusks, (b) infaunal mollusks, (c) epifaunal mollusks, and (d) all species. The boundaries of the box indicate the 25th and 75th percentiles. Error bars denote the 90th and 10th percentiles. The open square indicates the mean, and the midline of the box indicates the median. Bars labeled with different lowercase letters are significantly different (*p* < .05). Abbreviations of species names are presented according to Table 2

#### 2.4.2 Sediment accretion

Sediment accretion was measured using the marker‐horizon method around both the RSET benchmarks and the transect sampling sites. Marker horizons around RSET benchmarks were deployed in July 2016 and were sampled at 3–12 month intervals until December 2018, comprising a total of six sets of measurements; the total period assessed was 29 months. Marker horizons around the transect sampling sites were deployed in July 2017 and were sampled at 3–6 month intervals until August 2019, comprising a total of four sets of measurement; the total period assessed was 25 months. Three feldspar markers (50 cm × 50 cm) were deployed around each sampling site or RSET benchmark. At each sampling time, one cubic soil core was taken at an undisturbed location on each marker horizon, and the depth of the sediment above the marker horizon at three positions in each core was measured to give an average reading per marker horizon. The mean depth across the three cores was used to represent sediment accretion at each sampling site or RSET benchmark.

### 2.5 Model building

We aimed to build dynamic models to predict surface elevation changes at different vertical positions in response to various rates of SLR, considering tidal inundation and sediment accretion. The models developed herein were based on two assumptions: First, there is a positive correlation between surface elevation change and sediment accretion in the intertidal zone (Fu et al., 2019; Lovelock et al., 2015); and second, rates of surface elevation change vary among elevations (Fu et al., 2018; Kolker et al., 2010). There is ecogeomorphic feedback between tidal inundation and sediment accretion, such that increases in tidal inundation will increase sediment elevation, in turn decreasing tidal inundation (Krauss et al., 2014; McKee et al., 2007; Morris et al., 2002).

To build the model, we first established a relationship between the rate of surface elevation change and sediment accretion. Second, we converted sediment accretion rates at the transect sampling sites into surface elevation change rates to establish a relationship between surface elevation change rate and surface elevation. Third, we used various rates of SLR to calculate yearly surface elevation changes at different elevations. The simulation time of the model was set to 2020–2100 to improve prediction accuracy (Breithaupt et al., 2018), and model simulations were based on data from the SJ transect, as elevation sampling was most complete along this transect. Because there is a seawall at the landward edge of the mangrove forest at our study site, mangroves are unable to migrate landward in response to SLR. The model was thus restricted to the current forest zone.

According to tide data provided by local tide‐gauge stations, the SLR rate in Zhenzhu bay was about 2.00 mm year^−1^ between 1980 and 2019 (SOA, 2020). It is predicted that global average SLR rate will accelerate in the future, increasing from 3.7 mm year^−1^ at present to 16.00 mm year^−1^ by 2100 (Church et al., 2013). Therefore, the SLR rate in our model was set between 2.00 mm year^−1^ and 16.00 mm year^−1^, with the extreme values used to explore the influence of the maximum and minimum SLR rates on the distributions of mangrove mollusks.

All model projections were performed using R 4.0.3 (R Development Core Team, 2011) (Fu et al., 2019), and the R code used is provided in Appendix S1.

### 2.6 Data analysis

Previous studies in the region found no significant seasonal changes in the vertical zonation patterns of mangrove mollusks (Ma et al., 2020). To compare vertical distributions among molluscan species, we first combined the data from four seasons and calculated the average density of each species at various elevations. We then standardized the density data for each species at various elevations by the total to makes the data comparable. Nonparametric Kruskal–Wallis tests, followed by stepwise step‐down comparisons, were used to compare vertical distributions among species. Quadratic regressions were used to investigate the relationship between mean vertical elevation and variance in vertical elevation across all species. Linear regressions were used to analyze trends in surface elevation change and the relationships between sediment accretion and surface elevation change at the RSET benchmarks. The empirical exponential decay function was used to describe the relationship between surface elevation change and surface elevation at the sampling sites. One‐sample Student's *t* tests were used to determine whether the surface elevation changes recorded using the RSET instruments differed significantly from those determined based on the ^210^Pb dating of sediment cores.

To predict the influence of SLR on mollusk abundance, we assumed that mollusk density varied linearly between adjacent sampling sites (25 cm elevation intervals), based on the correlation between mollusk distribution and surface elevation. Linear models of mollusk density and surface elevation were established between each pair of adjacent elevations. First, the horizontal distance between each pair of adjacent sampling sites was calculated, multiplied by the mean density of the paired sampling sites, and summed to represent original abundance. Second, various rates of SLR were used to calculate changes in surface elevation at each sampling site. Mollusk density at the new elevation was predicted using the linear model of mollusk density and surface elevation, and the mean density of each pair of adjacent sampling sites was multiplied by the horizontal distance and summed to represent the new abundance. If the new elevation of the sampling site was less than the minimum elevation for mangrove survival (−15 cm), species abundance was calculated based on the horizontal distance between the location of the minimum elevation and the paired sampling site. Finally, the change in mollusk abundance was calculated by comparing the predicted new abundance to the original abundance.

All analyses were performed using SPSS v26.0 (IBM, 2019). Line charts, box plots, 3D wire‐frame plots, and 3D bars plots were generated using Origin v9.8.0 (OriginLab, 2020).

## 3 RESULTS

### 3.1 Molluscan species distributions

Across all sampling sites (elevations of −15 cm to 150 cm), we collected 191,130 mollusks, which we assigned to 39 species (Table 2). The epifaunal mollusks were the most species rich, with 38,995 individuals from 23 species. We collected 149,018 arboreal mollusks from eight species and 3117 infaunal mollusks from eight species. Mollusks of each assemblage type were distributed throughout the elevation range. Molluscan vertical distributions exhibited obvious patterns of zonation correlating with surface elevation: There were significant differences in elevation distributions among species, regardless of assemblage type (*p* < .05; Figure 2). For example, *Littoraria scabra* had the lowest vertical distribution among arboreal species (mean elevation, 2.25 cm). More than 80% of all *L. scabra* individuals were found between −15 cm and 10 cm, significantly lower than the infaunal mollusks *Geloina coaxans* and the arboreal mollusks *Cerithidea ornata* (mean elevations, 129.70 cm and 137.30 cm, respectively; *p* < .05). The vertical ranges of several species overlapped. For example, the vertical distribution of *L. scabra* did not differ significantly from that of the epifaunal mollusk *Batillaria multiformis* (mean elevation, 3.50 cm; *p* > .05). Although these species have differently life history modes, both inhabit the seaward edge of the mangrove forest.

**TABLE 2:** Abbreviation and assemblage type of mollusks

Scientific Name	Abbreviation	Assemblage
*Littoraria scabra*	*Ls*	Arboreal
*Enigmonia aenigmatica*	*Ea*	Arboreal
*Littoraria pallescens*	*Lp*	Arboreal
*Littoraria melanostoma*	*Lm*	Arboreal
*Littoraria ardouiniana*	*Lar*	Arboreal
*Terebralia sulcata*	*Ts*	Arboreal
*Cerithidea ornata*	*Co*	Arboreal
*Cassidula nucleus*	*Cn*	Arboreal
*Merisca capsoides*	*Mc*	Infaunal
*Pinguitellina cycladifomis*	*Pc*	Infaunal
*Laternula anatina*	*La*	Infaunal
*Glauconome chinensis*	*Gc*	Infaunal
*Indoaustriella scarlatoi*	*Is*	Infaunal
*Indoaustriella plicifera*	*Ip*	Infaunal
*Cerithidea microptera*	*Cm*	Infaunal
*Geloina coaxans*	*Gco*	Infaunal
*Nassarius nodifer*	*Nn*	Epifaunal
*Nassarius sinarus*	*Ns*	Epifaunal
*Batillaria zonalis*	*Bz*	Epifaunal
*Cerithidea cingulata*	*Cc*	Epifaunal
*Clithon faba*	Cf	Epifaunal
*Nerita yoldi*	*Ny*	Epifaunal
*Batillaria multiformis*	*Bm*	Epifaunal
*Patelloida pygmaea*	*Pp*	Epifaunal
*Cerithidea djadjariensis*	*Cd*	Epifaunal
*Assiminea brevicula*	*Ab*	Epifaunal
*Iravadia quadrasi*	*Iq*	Epifaunal
*Onchidium struma*	*Os*	Epifaunal
*Assiminea latericea*	*Al*	Epifaunal
*Mainwaringia dantaae*	*Md*	Epifaunal
*Laemodonta octanfracta*	*Lo*	Epifaunal
*Salinator sanchezi*	*Ss*	Epifaunal
*Iravadia* sp.	*Isp*	Epifaunal
*Cerithidea largillierti*	*Cl*	Epifaunal
*Pharella acutidens*	*Pa*	Epifaunal
*Neritina violacea*	*Nv*	Epifaunal
*Stenothyra japonica*	*Sj*	Epifaunal
*Allochroa layardi*	*Ala*	Epifaunal
*Iracadia sakaguchii*	*Isa*	Epifaunal

**FIGURE 3:**
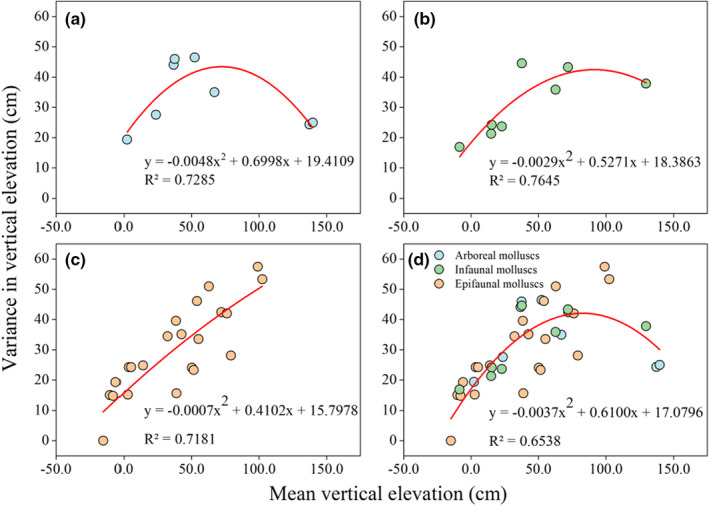
Relationships between mean vertical elevation and variance in vertical elevation of (a) arboreal mollusks, (b) infaunal mollusks, (c) epifaunal mollusks, and (d) all species

Across all assemblages, there was a significant quadratic relationship between vertical elevation variance and mean elevation (*R* ^2^ = 0.6538, *p* < .001, Figure 3d). Variance in molluscan vertical distributions increased and then decreased as mean elevation increased, with the highest variance observed at a mean elevation of 81.58 cm. We also identified significant quadratic relationships between vertical elevation variance and mean elevation, specifically, for arboreal mollusks (*R* ^2^ = 0.7285, *p* = .038, Figure 3a) and infaunal mollusks (*R* ^2^ = 0.7645, *p* = .027, Figure 3b), with the highest variance observed at mean elevations of 72.90 cm and 90.88 cm, respectively. For epifaunal mollusks, there was a significant quadratic relationship between vertical elevation variance and mean elevation (*R* ^2^ = 0.7181, *p* < .001, Figure 3c), but the variance in vertical elevation increased steadily with mean elevation. Compared to arboreal and infaunal mollusks, epifaunal mollusks inhabiting high elevations have wider distribution among elevations and might be more adaptable to SLR.

### 3.2 Changes in surface elevation and sediment accretion

**FIGURE 4:**
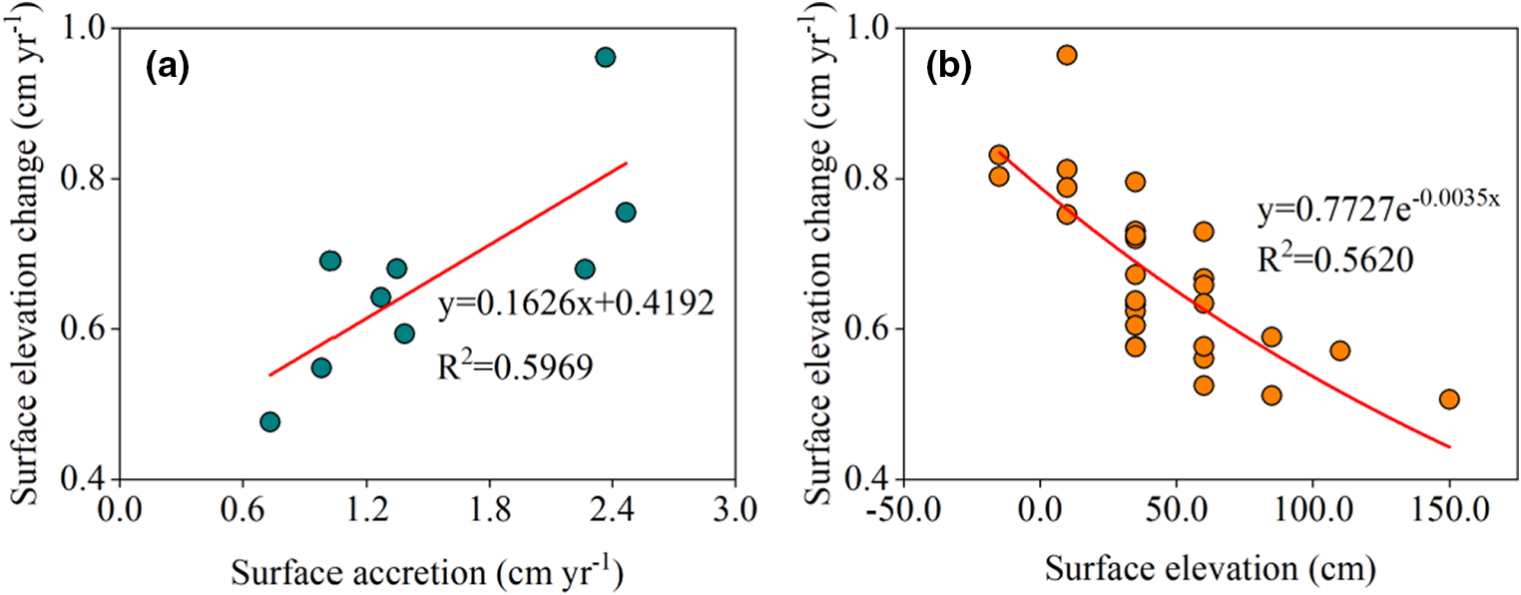
Relationships between surface elevation change and (a) surface accretion, (b) surface elevation

Over the study period, surface elevations at the RSET benchmarks increased significantly (*p* < .001), with an average increase of 6.70 mm year^−1^ (ranging from 4.76 to 9.61 mm year^−1^). Due to tidal erosion and bioturbation, the horizon markers at the RSET benchmarks in the *A. corniculatum* forest only provided continuous sediment‐accretion data for 1 year (2017–2018). The surface accretion at the RSET benchmarks was 7.35–24.67 mm year^−1^, which was proportional to the change in surface elevation (*R* ^2^ = 0.5969, *p* = .015; Figure 4).

**FIGURE 5:**
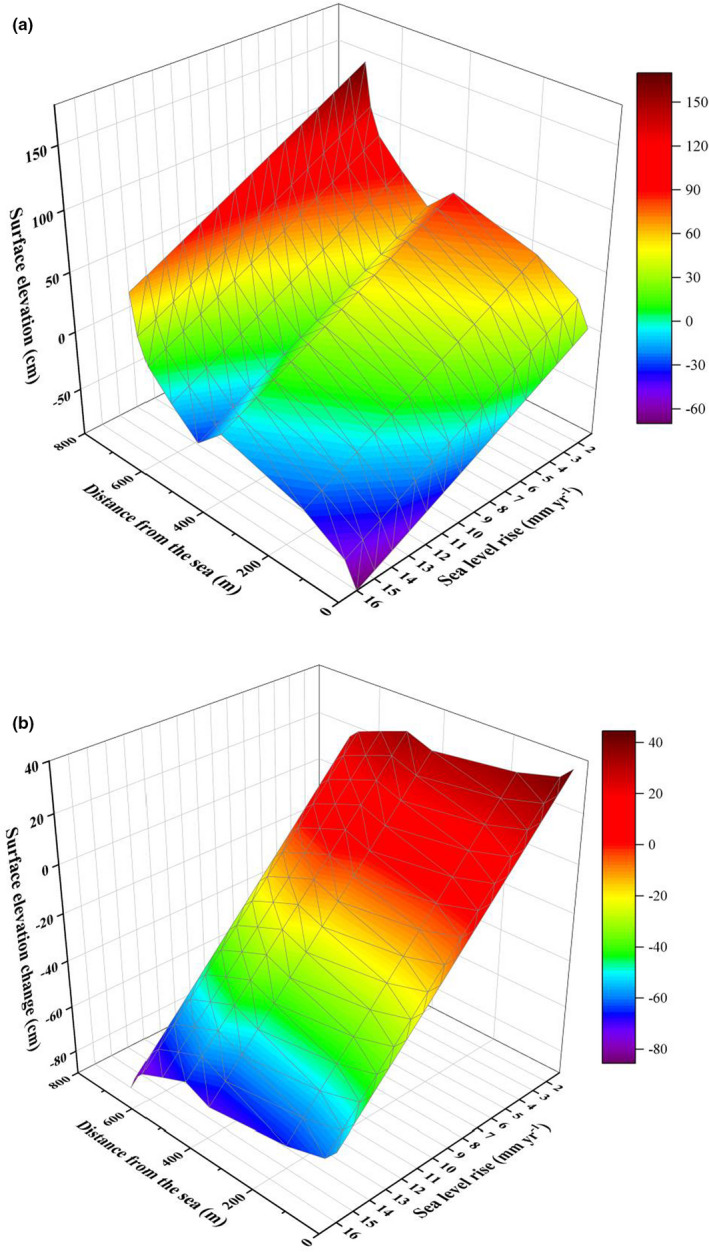
Model‐based predictions of (a) surface elevation and (b) surface elevation change in the year 2100 assuming different degrees of sea level rise

Using the linear relationships between surface elevation change and sediment accretion at the RSET benchmarks, we calculated surface elevation change at the transect sampling sites based on the sediment accretion rates. Across all markers, surface elevation change decreased exponentially as surface elevation increased (Figure 5); that is, the surface elevations of seaward sites changed more than the surface elevations of landward sites.

### 3.3 Dynamic changes in surface elevation and mollusk species distributions

Our model predicted that changes in surface elevation would decrease as the original surface elevation increased, and that surface elevation would first increase and then decrease as SLR increased (Figure 5). At low rates of SLR (2.00–4.57 mm year^−1^), the changes in surface elevation invariably outstripped the rate of SLR, and the model suggested that surface elevation would increase at all sites by 2100. At moderate rates of SLR (4.57–8.14 mm year^−1^), the model predicted that surface elevation would begin to decrease at the landward sites and gradually spread to seaward sites as the rate of SLR increased. At high rates of SLR (8.14–16.00 mm year^−1^), SLR was greater than the changes in surface elevation at all sites, and the model suggested that surface elevation would decrease at all sites by 2100.

**FIGURE 6:**
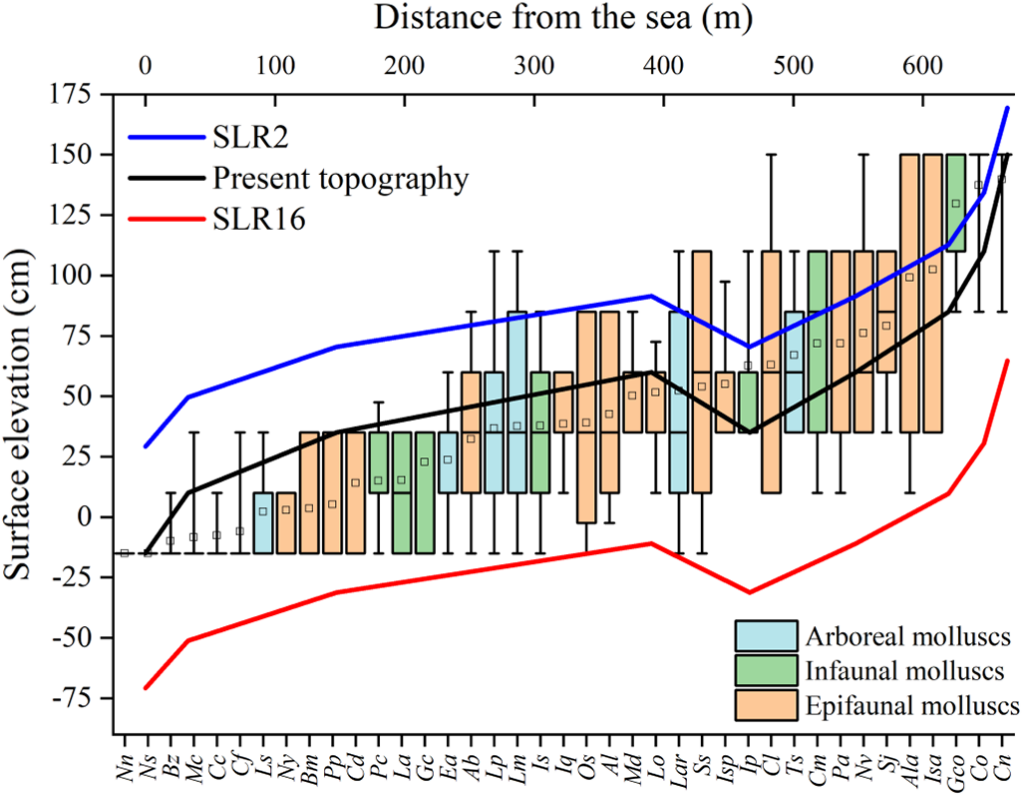
Current mollusk species distributions overlain on simulated topography of the intertidal zone in 2100 given maximum and minimum sea level rise. The boundaries of the box indicate the 25th and 75th percentiles. Error bars denote the 90th and 10th percentiles. The open square indicates the mean, and the midline of the box indicates the median. Legend abbreviations: SLR2, topography of the intertidal zone in the year 2100, as simulated by the model with a sea level rise (SLR) under 2 mm year^−1^; SLR16, topography of the intertidal zone in the year 2100, as simulated by the model with an SLR under16 mm year^−1^. Abbreviations of species names are presented according to Table 2

We plotted the predicted changes in intertidal topography given the two extremes of SLR (2.00 mm year^−1^ and 16.00 mm year^−1^) over the current intertidal profile and molluscan zonation patterns (Figure 6). At the lowest SLR, we predicted that the mangrove forests would expand into the seaward intertidal zone due to the increase in surface elevation at all sites by 2100. Meanwhile, reductions in tidal flooding will result in the migration of mollusks with specific adaptations to the intertidal zone to lower surface elevations. Furthermore, a consequent increase in interspecies competition will further exacerbate the migration. Species presently inhabiting the seaward forest zone, such as *Nassarius semiplicatus*, *Batillaria zonalis*, and *Cerithidea cingulata*, will follow the mangroves and migrate to suitable habitats lower in the seaward intertidal, while their present habitats may be invaded by higher‐intertidal species such as *Assiminea brevicula* or *Assiminea latericea*. In addition, species presently inhabiting the landward forest, such as *C. ornata* and *Cassidula nucleus*, may experience population increases and range expansions due to the increase in upper‐intertidal habitats.

At the highest SLR, we predicted that surface elevations would decrease at all sites by 2100, suggesting that the percentage of sites at suitable elevations for mangrove growth along the simulated transect would be greatly reduced: The 2100 mangrove zone will be 29.6% as wide as the 2020 mangrove zone. In addition, mangrove forests on the seaward edge of the zone would disappear, and the slope of the mangrove forest on the landward edge would become steeper. Mollusk habitats would consequently be restricted to a smaller range of elevations (−15.00–64.69 cm), suggesting that molluscan density loss will increase with original mean elevation. That is, mollusks inhabiting lower‐intertidal zones in 2020 will migrate landward, invading the habitats of species at higher elevations. However, because the seawall prevents the landward expansion of the mangrove forest, mangrove mollusks in higher mean elevations, such as *C. ornata* and *C. nucleus*, will be unable to migrate further landward and may thus be outcompeted by lower‐intertidal species more well‐suited to the increases in tidal flooding. Thus, these upper‐intertidal communities may be lost.

**FIGURE 7:**
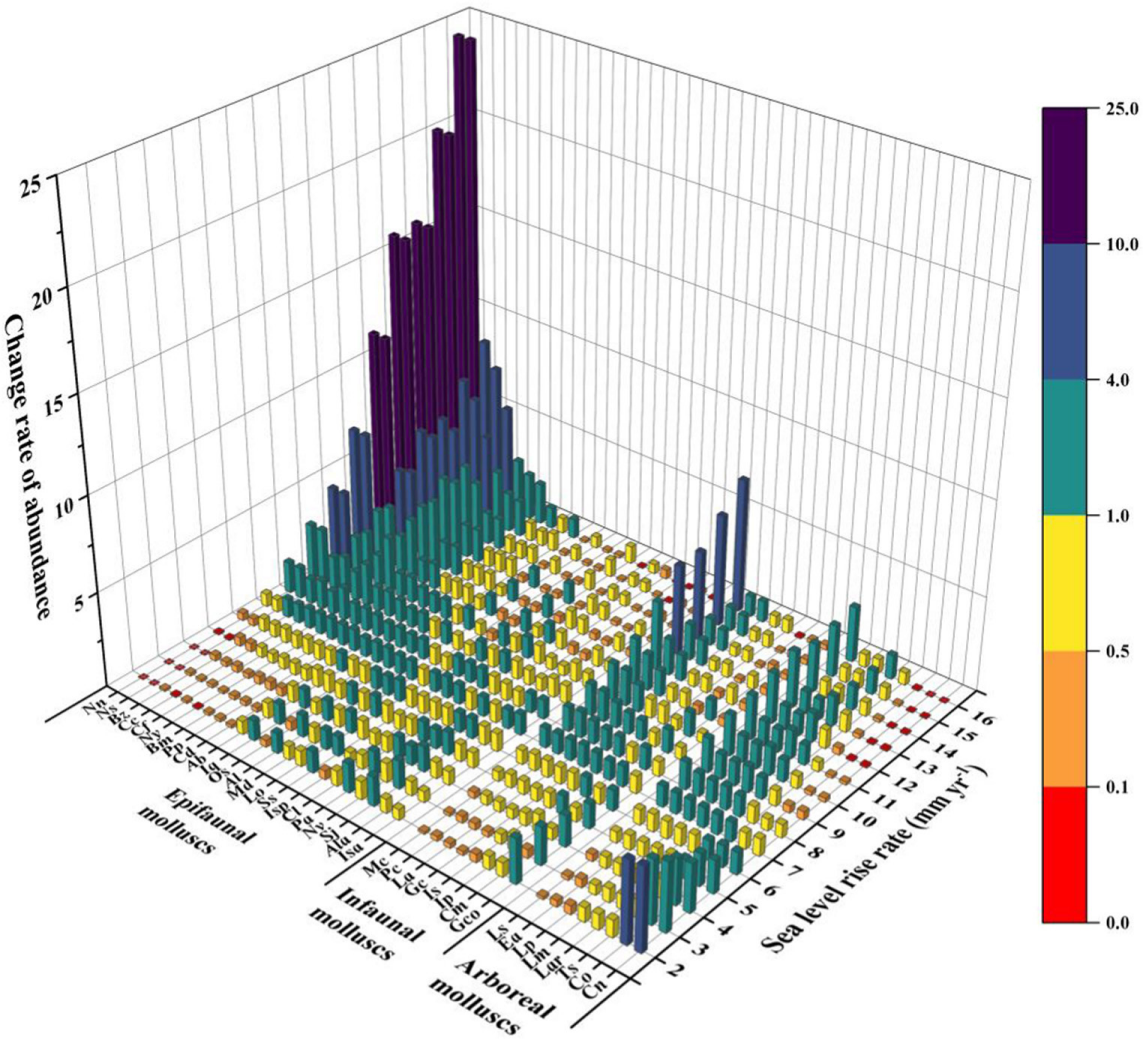
Predicted change rate of mollusk abundance in 2100 compared to 2020 abundance at various sea level rise rates. Abbreviations of species names are presented according to Table 2

**FIGURE 8:**
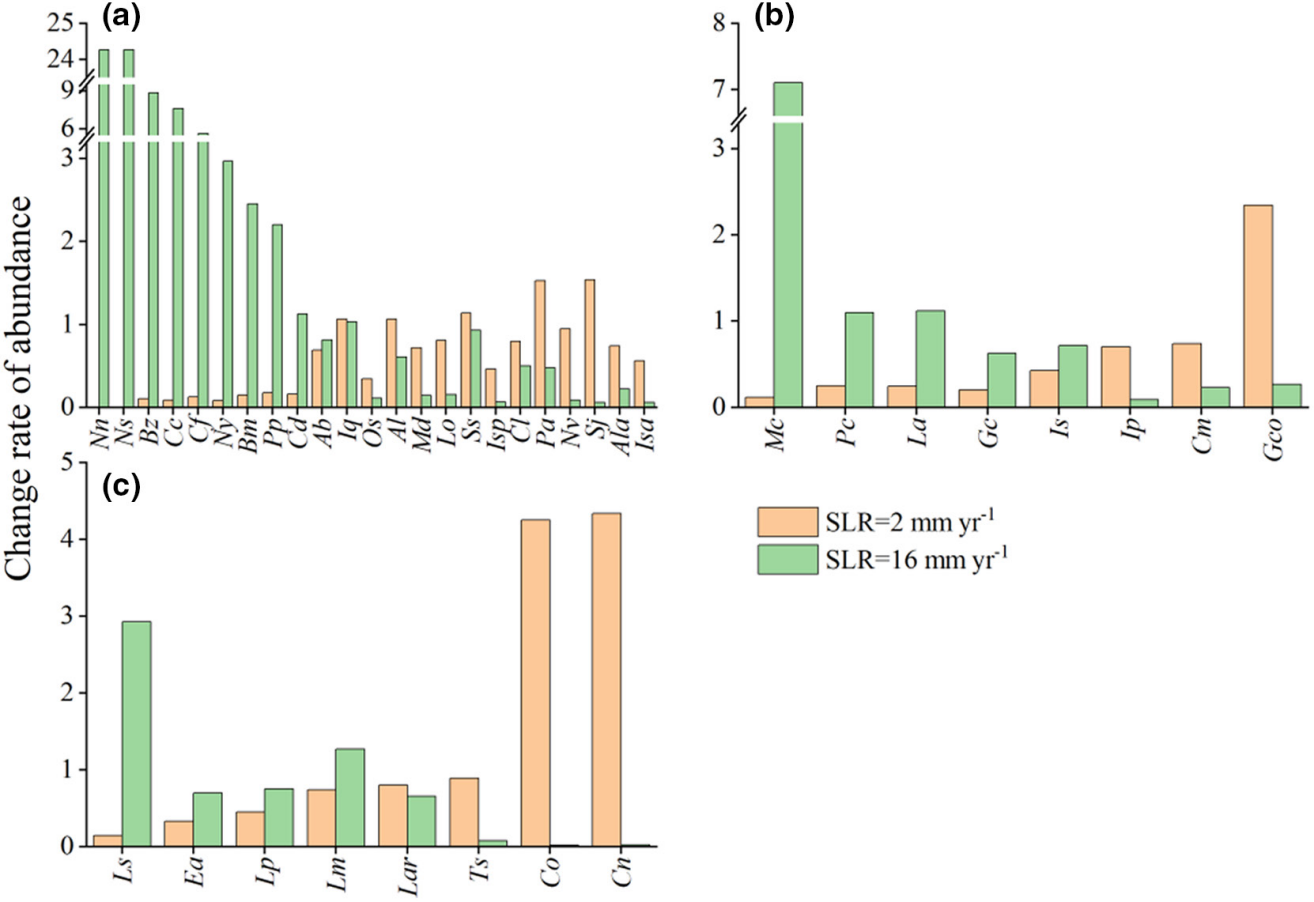
Predicted change rate of mollusk abundance in 2100 compared to 2020 abundance under two SLR (sea level rise) scenarios (2 and 16 mm year^−1^) of (a) epifaunal mollusks, (b) infaunal mollusks and (c) arboreal mollusks. Abbreviations of species names are presented according to Table 2

Our predictions of SLR‐driven changes in molluscan abundance indicated that the abundance of species in low elevations would decrease at low rates of SLR (Figure 7). The models predicted that species inhabiting low elevations would expand into the seaward intertidal zone due to the increases in surface elevation associated with low rates of SLR, and the abundance of these species would decrease within the current forest zone. The abundance of species inhabiting low elevations would increase with increased SLR, especially epifaunal species on the seaward edge of the forest. For example, at the highest SLR, the abundance of the epifaunal species *Nassarius nodifer* would be 2429% of its present abundance, while the abundance of the arboreal species *L. scabra* would be 293% of its present abundance (Figure 8a and c). For species inhabiting high elevations, abundance was predicted to be highest at the lowest rates of SLR and was predicted to decrease as SLR increased. For example, the abundance of *C. ornata* would increase to 425% of present abundance at a low rate of SLR (2.00 mm year^−1^) but would decrease to 1.79% of present abundance at a high rate of SLR (16.00 mm year^−1^) (Figure 8c). At other elevations, molluscan abundance was predicted to first increase and then decrease as SLR increased, but these changes were predicted to be relatively small compared with species on the seaward or landward edge of the forest. For arboreal and infaunal mollusks, the rates of changes in species abundance would increase with increasing distribution elevation of species at low SLR and decrease with increasing distribution elevation of species at high SLR. For epifaunal mollusks, species abundance was only related to distribution elevation of species at the seaward edge of the forest. The relationship between species abundance and distribution elevation of epifaunal mollusks inhabiting high elevations was not obvious, as their distribution range increased with increasing distribution elevation. Therefore, the abundance of epifaunal species inhabiting high elevations was predicted to decrease less than the abundances of arboreal and infaunal mollusks at high SLR.

## 4 DISCUSSION

### 4.1 Changes in surface elevation

Over the study period (2016–2020), surface elevations at all RSET benchmarks increased significantly (6.70 mm year^−1^, on average). These increases were greater than the worldwide average for pristine mangrove forests (0.70 mm year^−1^, Sasmito et al., 2016), but similar to surface elevation changes measured using RSET benchmarks in China and Vietnam (9.60 and 6.22 mm year^−1^, respectively; Fu et al., 2018; Lovelock et al., 2015). This indicated that the mangrove forests of the South China Sea had in some ways adapted to SLR. The surface elevation change rates obtained in this study did not differ significantly from the 122‐year sediment accretion rate in the same area determined using ^210^Pb (1888–2010; 6.70 mm year^−1^; Li, 2010) (*p* = .993), which indicated the reliability of the RSET estimations.

Changes in surface elevation in coastal wetlands are affected by several factors, including SLR, inundation duration, plant types, and sediment supply (Cahoon & Reed, 1995; Fu et al., 2019; Krauss et al., 2014; Woodroffe et al., 2016). At the RSET benchmarks used in this study, surface elevation changes were significantly correlated with sediment accretion, showing that mangrove forests have the capacity to increase surface elevation via the vertical accretion of sediments. This finding was consistent with other studies of the Indo‐Pacific region, which suggests that sediment supply plays an important role in the increase of surface elevation (Lovelock et al., 2015).

Changes in surface elevation in the intertidal, calculated based on sediment accretion, decreased with ground elevation, reflecting the dynamic, ecogeomorphic feedback between tidal inundation and surface elevation change (Kirwan et al., 2016). Studies have shown that the increased frequency and duration of tidal inundation in the lower intertidal increase the rates of mineral sediment and organic matter accumulation (Kirwan et al., 2016; Lovelock et al., 2011; Rogers et al., 2005; Woodroffe et al., 2016). However, earlier static landscape models projected SLR onto a static topography with a constant historical rate of elevation change, ignoring the ecogeomorphic feedback that allows intertidal ecosystems to adapt to sea level changes and thus overestimated the impact of sea level changes (Di Nitto et al., 2014; Kirwan et al., 2016). Compared to traditional static models, dynamic models that include the ecogeomorphic feedback between surface elevation and surface elevation change better simulate the impact of sea level change on different vertical positions in the intertidal zone and better predict the adaptability of the intertidal zone to SLR (Fagherazzi et al., 2012; Fu et al., 2019; Kirwan et al., 2010; Kirwan et al., 2016).

### 4.2 Impact of SLR on the distributions of mangrove mollusks

Mangrove mollusks in Zhenzhu Bay exhibited distinct zonation patterns, with certain species occurring at specific elevations; these zonation patters were typically related to tidal inundation and mangrove species abundance (Alfaro, 2006; Alongi, 2009; Ma et al., 2020; Reid, 1985). SLR was predicted to affect molluscan distributions and change molluscan community structure, but the predicted effects of SLR varied based on species assemblage and vertical distribution.

Our models suggested that lower‐intertidal mollusks on the seaward side of the forest would be less affected by SLR, as suitable habitats will not disappear even in the most extreme SLR scenarios. In addition, these species are known to migrate naturally at different life stages. For example, *C. cingulata*, a common mollusk on the seaward edges of mangrove forests and adjacent mudflats in Southeast Asia (Reid et al., 2008), migrates to higher elevations as it ages (Yang & Shen, 1992). This migration may reflect a preference for sediment size at different life stages (Vohra, 1970).

Based on our model predictions, mollusks that inhabit the central mangrove forest will be more affected by SLR than those at the seaward forest edge because of their wider distribution among elevations, and the response to SLR is likely to differ among species. Mollusks in the central mangrove forest rarely leave their initial colonization site, and the distributions of these species thus exhibit long‐term spatial inertia (Vannini et al., 2008). In particular, arboreal mollusks in the genus *Littoraria* spend their entire lives on mangrove plants, moving up and down the trunk between tides to forage (Reid, 1986). They can only adapt to SLR by breeding new individuals to migrate to suitable elevation. Most species of *Littoraria*, such as *Littoraria melanostoma*, are oviparous, moving down the trunk to release eggs into the seawater below (Reid, 1989; Reid, 1992). However, other littorinids, such as *Littoraria ardouiniana*, are ovoviviparous, releasing planktotrophic veliger larvae into seawater after a short brooding period; because larvae are released quickly, ovoviviparity carries a lower underwater predation risk than ovipary (Ng & Williams, 2012; Reid, 1986; Reid, 1989). This reproductive strategy also allows *L. ardouiniana* to inhabit higher mean elevations than *L. melanostoma* because the former species requires briefer periods of tidal inundation (Lee & Williams, 2002), which was consistent with our findings. However, due to its higher mean elevation, *L. ardouiniana* is more vulnerable to SLR, as our model showed that the surface elevation of higher elevations will decrease with SLR. In addition, species with an ovoviviparous reproductive strategy may have narrower larval dispersal ranges than oviparous species because of the shorter time of larval duration (Berry & Chew, 1973; Reid, 1986; Reid, 1989). Thus, it may be more difficult for ovoviviparous larvae to colonize suitable elevations if local surface elevations change. Therefore, the effects of sea level change may differ among species in the same habitat, suggesting that future monitoring studies should not ignore patterns of molluscan larval dispersal.

One of the key characteristics of mollusks that inhabit landward mangrove sites in high elevations, such as the halophile ellobiids, is their intolerance of tidal inundation: Lengthy periods of inundation are fatal to such species, which lack an operculum and have lungs adapted to air‐breathing (Martins, 2001; Morton & Graham, 1955; Ragionieri et al., 2015). As SLR increases, our models predicted that landward sites would be the first to undergo elevation loss. Indeed, at the highest predicted rate of SLR, elevation at the highest site was predicted to decrease substantially, from the present 150 cm to 64.69 cm, effectively eliminating suitable habitats for upper‐intertidal mollusks, especially those of arboreal and infaunal mollusks with limited vertical ranges. In addition, greater predation risks due to increased tidal inundation and the intensification of interspecific competition brought about by the landward movement of lower‐intertidal species will result in large‐scale decreases or eliminations of the mollusks inhabiting the landward mangrove forest (Rochette & Dill, 2000).

Anthropogenic threats, including agri‐ and aquacultural development, pollution, and resource overextraction, are the proximate drivers of contemporary mangrove decline and degradation (Lee et al., 2006; Richards & Friess, 2016; Thomas et al., 2017). However, with the recognition of the ecosystem services provided by mangroves and the expansion of mangrove management and protection programs, annual mangrove loss rates decreased from ~2% to <0.4% between the late 20th century and the early 21st century (Friess et al., 2019). Despite these improvements in mangrove conservation, mangroves may become increasingly threatened due to future accelerations in SLR (Krauss et al., 2014; Lovelock et al., 2015). Effective ecosystem management and mangrove conservation programs are needed to support the adaptation of mangrove forests to SLR (Friess et al., 2020). Our results showed that the mangroves in Zhenzhu Bay will tolerate the rates of SLR currently predicted, and that suitable habitats for mangrove mollusks will remain, due to increases in surface elevation. However, the adaptive capacity of the Zhenzhu Bay mangroves to SLR is limited by two anthropogenic factors. First, our models suggested that, at high rates of SLR, the mangrove forests would migrate into the landward intertidal zone, followed by the mollusks. However, artificial topographical barriers will prevent landward mangrove movement, resulting in the loss of suitable habitat for mollusks restricted to higher elevations and thus overall decreases in molluscan biodiversity. Under accelerated SLR conditions, it will be necessary to protect not only current mangrove areas, but also potential future mangrove habitats to support mangrove adaption to environmental change (Friess et al., 2020; He & Silliman, 2019). Second, because rivers are a key source of sediments, river damming substantially reduces inshore suspended sediment concentrations, affecting mangrove surface accretion and reducing the resilience of the mangroves to SLR (Friess et al., 2019; Lovelock et al., 2015; van Wesenbeeck et al., 2014). Due to the important ecological functions of mangrove forests, river damming projects should be undertaken based on careful consideration of the downstream impacts on mangrove survival (Friess et al., 2020).

## 5 CONCLUSION

In this study, we predicted the effects of SLR on molluscan spatial distributions by constructing dynamic models of the relationship between SLR and changes in surface elevation. The rates of surface elevation change in Zhenzhu Bay were higher than the worldwide average in pristine mangroves, and the magnitude of change in surface elevation decreased exponentially as surface elevation increased. The dynamic models predicted that mangroves in Zhenzhu Bay will be able to adapt to current sea level changes (2.00 mm year^−1^) by 2100. Although the surface elevations of mangroves at high surface elevations were predicted to decrease if SLR rates continue to increase, our models did not predict total mangrove loss, even at the most extreme rates of SLR (16.00 mm year^−1^). Based on the results of our models, we predicted that mollusks on the landward edge of the mangrove forest, especially the arboreal and infaunal assemblages, would be more sensitive to SLR, and that the survival of these groups would be threatened both by the increased inundation duration associated with rapid SLR and by intensified interspecific competition due to coastal squeeze. Future ecological mangrove conservation efforts should consider potential species habitats to maintain the resilience of mangroves to SLR.

## AUTHOR CONTRIBUTIONS

**Wei Ma:** Conceptualization (equal); data curation (equal); formal analysis (equal); investigation (equal); methodology (equal); project administration (equal). **Mao Wang:** Conceptualization (equal); funding acquisition (equal); methodology (equal); project administration (equal). **Haifeng Fu:** Data curation (equal); investigation (equal); methodology (equal). **Chaoyi Tang:** Investigation (equal). **Wenqing Wang:** Conceptualization (equal); funding acquisition (equal); methodology (equal); project administration (equal).

## CONFLICT OF INTEREST

The authors declare no competing interests.

## Supporting information


Appendix S1

## Data Availability

The data used in this study have been archived through Dryad online data repository and are publically available at https://doi.org/10.5061/dryad.pg4f4qrr9
