# Supplementary material for: Predicting changes in molluscan spatial distributions in mangrove forests in response to sea level rise
Source: Ecol Evol. 2022 Jul 13;12(7):e9033. doi: 10.1002/ece3.9033 (PMC9277612; doi:10.1002/ece3.9033)
Supplement: Supplementary file 1 — Appendix S1 [file ECE3-12-e9033-s001.doc]

**Text 1. R codes for estimating the surface elevations relative to sea level along the intertidal profile with different sea level rise rates.**

for(SLR in 2:16){

elevation=c(-15,10,35,60,85,110,150)

for(t in 1:80){

rate=0.7727*exp(elevation*-0.0035)

rate=rate-SLR*0.1

elevation=elevation+rate

t=t+1}

print(elevation)

SLR=SLR+1

}
